# Accurate detection and density estimation of peach tree inflorescences using an improved YOLOv11 model

**DOI:** 10.3389/fpls.2026.1722418

**Published:** 2026-02-18

**Authors:** Jiangtao Ji, Xiaoxuan Lu, Hao Ma, Xinyi Lu, Yaqing Yang, Hongwei Cui, Meijia Yu, Xuran Xie

**Affiliations:** College of Agricultural Equipment Engineering, Henan University of Science and Technology, Luoyang, Henan, China

**Keywords:** deep learning, inflorescence detection, multi-scale feature fusion, peach tree, YOLOv11

## Abstract

Flower thinning plays a vital role in peach production, which significantly affects fruit yield and quality. Obtaining precise information about inflorescences is the key to scientific thinning and refined orchard management. However, the accurate detection of peach inflorescence still faces great challenges due to the complex and changeable light conditions, dense occlusion between flowers and significant scale differences in the actual orchard environment. In order to solve these problems, an enhanced YOLOv11s peach inflorescence detection model, termed MDI-YOLOv11, is proposed in this study to achieve accurate and stable recognition of flowers and buds. Considering the characteristics of small target and frequent occlusion in peach inflorescences, a collaborative design of the neck feature fusion structure and the backbone feature attention mechanism is adopted. Specifically, the RFCAConv module is added to the backbone network to increase sensitivity to salient regions, while a P2 layer for small target detection is embedded within the neck network and integrated with the RepGFPN structure to enhance multi-scale feature fusion, thereby improving detection accuracy and adaptability in complex orchard environments. The model’s performance was systematically assessed on a self-built dataset comprising 1,008 images. The dataset labeled 41,962 target instances after sample balancing, including 22,803 flower targets and 19,159 bud targets, covering typical orchard scenes with varying illumination, color characteristics, and high density occlusion. The five-fold cross-validation experiment demonstrated that MDI-YOLOv11 achieved an AP_50_ of 0.919 and an AR_50_ of 0.964 for peach tree inflorescences detection, along with a detection time of 13.46 ms per image. 10.97 million parameters, and a model size of 21.51MB, all of which meet practical application requirements. Compared with the YOLOv11s model, the MDI-YOLOv11 model achieved a 0.033 increase in both AP_50_ and AR_50_, and the detection performance and model complexity are better than YOLOv11m. Based on the detection results of MDI-YOLOv11, this study generated row-by-row inflorescence density distribution maps that intuitively displayed the spatial density distribution of peach inflorescences. The results indicate that the proposed method enables efficient and accurate detection of peach flowers and the generation of inflorescence density maps, which is expected to provide effective support for refined orchards management.

## 1 Introduction

Peach is the second most productive economic fruit tree globally, with a yield second only to apples. In 2023, China played a dominant role in the global peach industry, producing over 16 million tons, which accounted for approximately 70% of total global peach production (Manganaris et al., 2022). In peach cultivation management, the density of peach blossoms directly impacts fruit yield and quality (Barreto et al., 2019). Rational flower thinning helps to reduce uneven nutrient distribution caused by excessive fruit setting, inhibit tree weakness, enhance stress resistance while optimizing flower bud quality in the coming year (Sutton et al., 2020; Zhang et al., 2024). Accurate detection of inflorescences is essential for intelligent and quantitative management, improving flower thinning efficiency, and promoting high quality and high yield of peach.

Early inflorescence detection mainly depended on conventional image processing methods, which involved steps such as image preprocessing, feature extraction, and classification to distinguish inflorescences from the background. However, these methods depended on manually designed features, such as edge features (Guo et al., 2024; Hao et al., 2021), color features (Horton et al., 2017), and texture features (Aggelopoulou et al., 2011). These features are easily limited by environmental complexity and controllability in practical applications and cannot meet the needs of complex environments. As machine learning techniques advanced, conventional approaches, including cluster analysis (Guihong et al., 2023), were gradually applied to inflorescence recognition, but they still relied on manually selected features for model training, resulting in insufficient adaptability in complex environments.

Recent advances in convolutional neural network–based deep learning methods (Li et al., 2021) have made them a central research direction in inflorescence recognition, due to their capacity for automatic feature learning and their effectiveness in addressing the limitations of traditional approaches. Lin et al. (Lin et al., 2020) employed an improved Faster R-CNN algorithm to detect strawberry flowers under different shooting angles, distances, and lighting conditions, and the detection accuracy reached 0.861. Chen et al. (Chen et al., 2019) developed an automatic strawberry flowers detection system based on Faster R-CNN, which demonstrated an average accuracy of 0.841 when compared with manual counting results. In addition to two-stage detectors, the YOLO series of popular one-stage detectors has demonstrated significant application potential in inflorescence detection tasks (Wentao et al., 2023; Yifan et al., 2024). Xia et al. (Ye et al., 2022) introduced the Ghost module and BiFPN structure into the YOLOv5s algorithm for detecting pear flowers on single branches in horizontal trellis systems, achieving a detection accuracy of 0.913. The detection time required just 29 ms per image, and the model size was only 7.62 MB. Lyu et al. (Shilei et al., 2022) proposed an embedded citrus flower recognition sensing system, deploying a YOLOv4-CF object detection model based on cascaded fusion on an FPGA platform to achieve real-time detection of close-up citrus flowers. The detection accuracy reached up to 0.95, and the detection speed was 30 frames per second on the FPGA, significantly enhancing both detection accuracy and real-time performance. Shang et al. (Yuying et al., 2023) introduced ShuffleNetv2 and the Ghost module into the YOLOv5s model to construct a lightweight real-time detection model for apple flowers. Real-time detection trials on close-up apple flowers were conducted on a Jetson Nano B01 development board, with a detection accuracy of 0.918 and a processing speed of 2.48 frames per second. Wang et al. (Wang et al., 2025) developed the YO-AFD model for apple flower detection by incorporating residual mobile blocks and attention mechanisms into the YOLOv8n architecture. The proposed model shows strong detection performance in a complex natural orchard environment, with mAP_50_ value of 0.941 and an F1 score of 0.886, while maintaining a low average inference time of only 5.3 ms per image. Xia et al. (Xue et al., 2023) introduced an apple inflorescence detection model, MTYOLOX, adapted to various lighting conditions for whole-tree level apple inflorescence data. The average accuracy of the model reached 0.834, the average recall rate reached 0.933, and by generating the inflorescence density heat map, the spatial distribution of apple inflorescence on the whole tree was visually displayed. Geng et al. (Geng et al., 2025) proposed a framework that integrates YOLOv8n-based object detection with an improved Density Peak Clustering (DPC) algorithm. In this approach, individual flower are first localized using the YOLOv8n model, and then the improved DPC method is used to cluster the detection result, automatically identify the inflorescence cluster and determine the positions of central flower, thereby effectively reducing localization deviations of the central flower. Sun et al. (Sun et al., 2024) introduced a YOLOv5-based peach inflorescence detection model in which the K-means++ algorithm was employed to optimize anchor box sizes. The improved model resulted in accuracy increases of 0.078, 0.101, and 0.034 for detecting peach buds, blossoms, and fallen flowers, respectively. Furthermore, Kun et al. (Kun et al., 2022) overcame the limitations of traditional RGB images and proposed the FC-Net flower counting model based on an RGB-D camera. This model leverages depth information to filter out interference from complex backgrounds and distant flowers, and enhances the network’s feature representation capabilities through the integration of attention and multi-scale feature fusion modules. It achieved a counting error rate of 0.02% in the peach flower counting task, providing a new direction for estimating peach flower counts in natural environments.

From the aforementioned studies, it can be seen that deep learning has shown significant potential in inflorescence detection and has made considerable progress. However, research on peach inflorescence detection remains limited, with most existing studies focusing on close-up inflorescence detection while neglecting the complexity of real-world peach orchard environments. In actual peach orchards, the overlapping branches, dense arrangement of flowers and complex shape bring challenges to the detection. Missed detections resulting from dense occlusion and scale variations in inflorescences are inadequately addressed by existing methods. Juan et al. (Sebastian et al., 2024) addressed the challenge of high-density object counting by proposing a density map prediction method based on multi-column convolutional neural networks, combined with color filtering to suppress background noise. The total number of flowers was then estimated by summing the pixel values of the density map. However, this approach still exhibited substantial errors in regions with high-density overlapping flowers, with MAE and RMSE reaching 19.13 and 69.69, respectively. These results indicate that dense occlusion remains a critical factor affecting the accuracy of flower counting. To further improve the generalization ability of peach tree inflorescence detection methods, this study proposes a multi-scale dense peach tree inflorescence detection model, MDI-YOLOv11. The key contributions of this paper are summarized below: (1) A two-stage object-level data balancing strategy is proposed, and a peach inflorescence dataset comprising 1,008 images and 41,962 target instances is constructed, which provides sufficient training data and effective class balance for the peach inflorescence detection model. (2) Combining the YOLOv11s model with the RepGFPN structure and RFCAConv module, a peach inflorescence detection model suitable for complex orchard scenes is developed. (3) Based on the detection results, row-structured inflorescence density heatmap is generated to visually represent the spatial distribution of peach tree inflorescences, providing scientific decision-making support for precise flower thinning.

## 2 Materials and methods

### 2.1 Dataset construction

#### 2.1.1 Data acquisition

**Figure 1:**
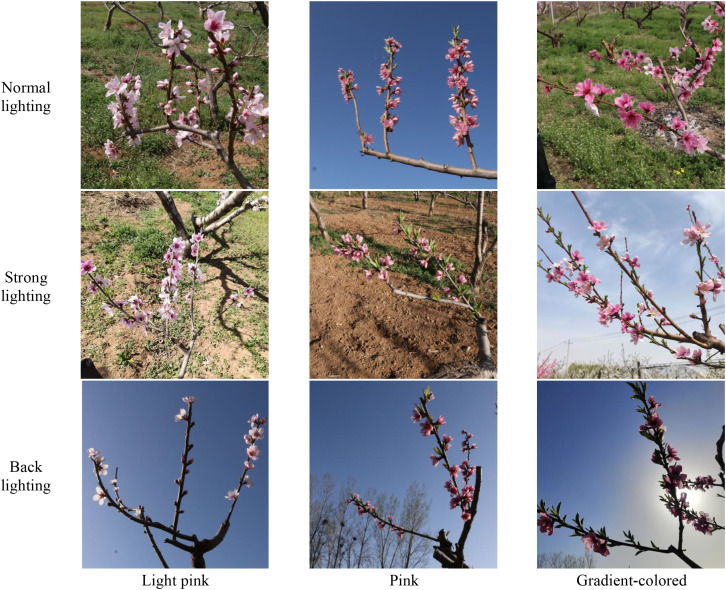
Peach flowers of different colors under varied lighting conditions.

The peach tree inflorescence images for this study were collected from Yangfeng Ecological Park in Mengjin County, Luoyang City, Henan Province, and the Luoyang Nongfeng Agricultural Technology Demonstration Park. The selected cultivars included ‘Chunmi’, ‘Chunxue’, and ‘Yanzhi Cuitao’. The collection time is from mid-March to early April 2024, which is the peak blooming period of peach trees. During this period, there were both fully blooming flowers and partially opened buds, which provided favorable conditions for inflorescence recognition. The images were captured using smartphones with resolutions of 4000×4000 and 3456×3456 pixels. The shooting distance is 30 to 80 centimeters by hand. The shooting time was from 9:00 a.m. to 12:00 p.m. and from 2:00 p.m. to 6:00 p.m. The dataset comprises a total of 1008 images, covering various complex scenarios, including different lighting conditions, flower colors, weather variations, and dense occlusions. **Figure 1** illustrates representative images of light pink, pink, and gradient-colored peach flowers captured under different illumination conditions. Under normal lighting, the images illumination is relatively uniform, the brightness is moderate, and the structural and textural features of the target regions remain stable. Under strong lighting, direct sunlight or high-intensity background light may lead to local brightness saturation, which partially affects the presentation of target details. In the backlight scene, the light source is positioned behind the target, the overall brightness of the target area is reduced, resulting in a decrease in the contrast between the foreground and the background, and usually accompanied by shadow enhancement or contour blur.

The image annotation was completed using the LabelImg tool, covering two categories: ‘peach_blossom’ and ‘peach_bud’, with a total of 36507 instances. To ensure the reliability of model evaluation and maximize the utilization of the dataset, a five-fold cross-validation strategy (Kohavi, 1995) was adopted. The dataset was evenly divided into five subsets, with each subset serving as the validation set in one iteration while the remaining four subsets were used for training. Five independent training sessions were conducted, and the overall model performance was evaluated based on the average results across the five training runs. The sample distribution for the five-fold cross-validation is presented in **Table 1**.

**Table 1:** Sample distribution for five-fold cross-validation.

Fold	Dataset (1008 images)				
	Group1 (202 images)	Group2 (202 images)	Group3 (202 images)	Group4 (201 images)	Group5 (201 images)
1	Test	Train	Train	Train	Train
2	Train	Test	Train	Train	Train
3	Train	Train	Test	Train	Train
4	Train	Train	Train	Test	Train
5	Train	Train	Train	Train	Test

#### 2.1.2 Data balancing

The peach tree inflorescence dataset consists of two categories, blossoms and buds, with a total of 36507 target instances. The number of blossom instances is approximately four times greater than that of bud instances (as shown in **Table 2**), resulting in a significant class imbalance. This class imbalanced may result in blossoms and buds to contributing unevenly to the loss function, causing the model to focus more on features of the majority class and consequently affecting its classification performance (Pablo et al., 2023; Solimani et al., 2024). For instance, the model may incorrectly classify the background as blossoms and exhibit missed or false detections for the bud category.

**Table 2:** Number of instances per class before and after data balancing.

Class	Original dataset	After the first stage	After the second stage
Peach_blossom	29034	29034	22803
Peach_bud	7473	12928	19159
All	36507	41962	41962

**Figure 2:**
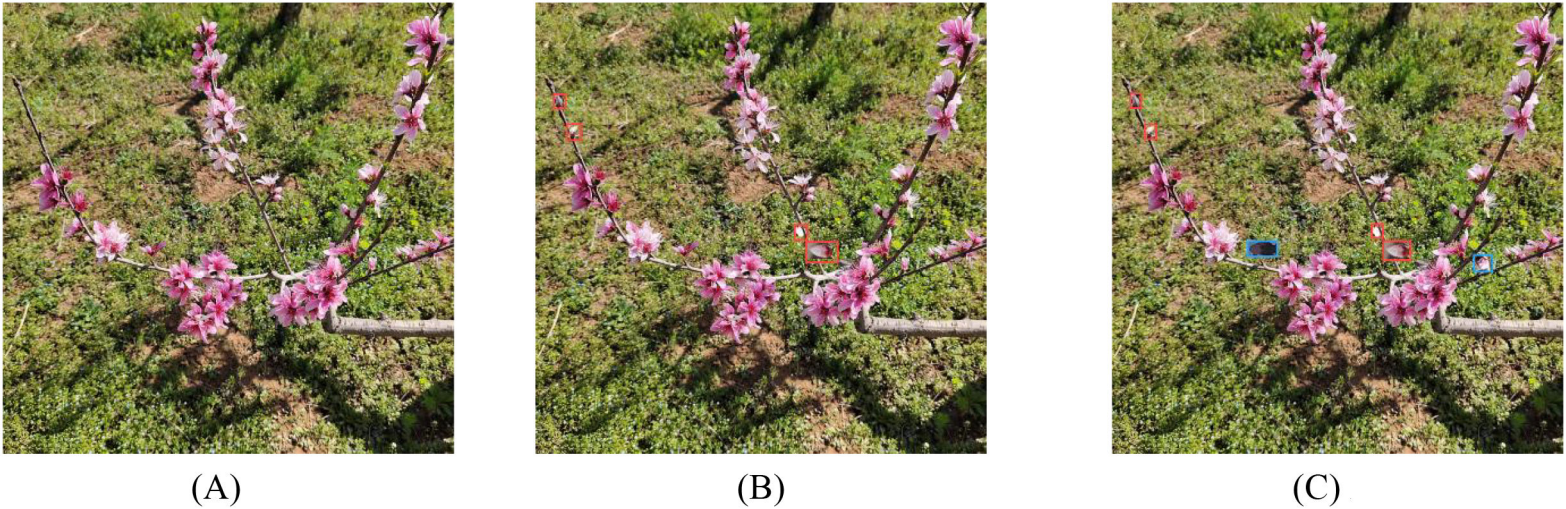
Image samples before and after data balancing: **(A)** Original image; **(B)** after the first stage; **(C)** after the second stage. In the figure, the red box indicates the target processed in the first stage, and the blue box indicates the target processed in the second stage.

When balancing the class distribution of the peach tree inflorescence dataset, it is essential to consider not only the differences in target quantity but also their spatial distribution. For example, peach flowers should appear on branches rather than on the ground or in midair. To address this, this study proposed a two-stage data balancing method, consisting of the following steps: (1) Extract bud instances from the original dataset and overlay them onto tender shoots after applying random transformations, ensuring that their spatial positions are reasonable and do not occlude other targets. (2) Extract bud instances and find flower targets with the closest aspect ratio of the bounding box that do not overlap with other target boxes, then overlay the transformed buds onto these flower target boxes. **Figure 2** presents a comparison of samples before and after data balancing. **Figure 2A** shows the image before balancing, while **Figures 2B, C** display the images after the two-stage processing. It can be observed that the newly added bud targets are appropriately arranged near branches and maintain a suitable scale. Considering the characteristics of inflorescence dataset, after the balancing process, the number of target instances increased to 41962, and the flower-to-bud ratio was adjusted from the original 4:1 to 1.2:1, as shown in **Table 2**.

### 2.2 YOLOv11 model

**Figure 3:**
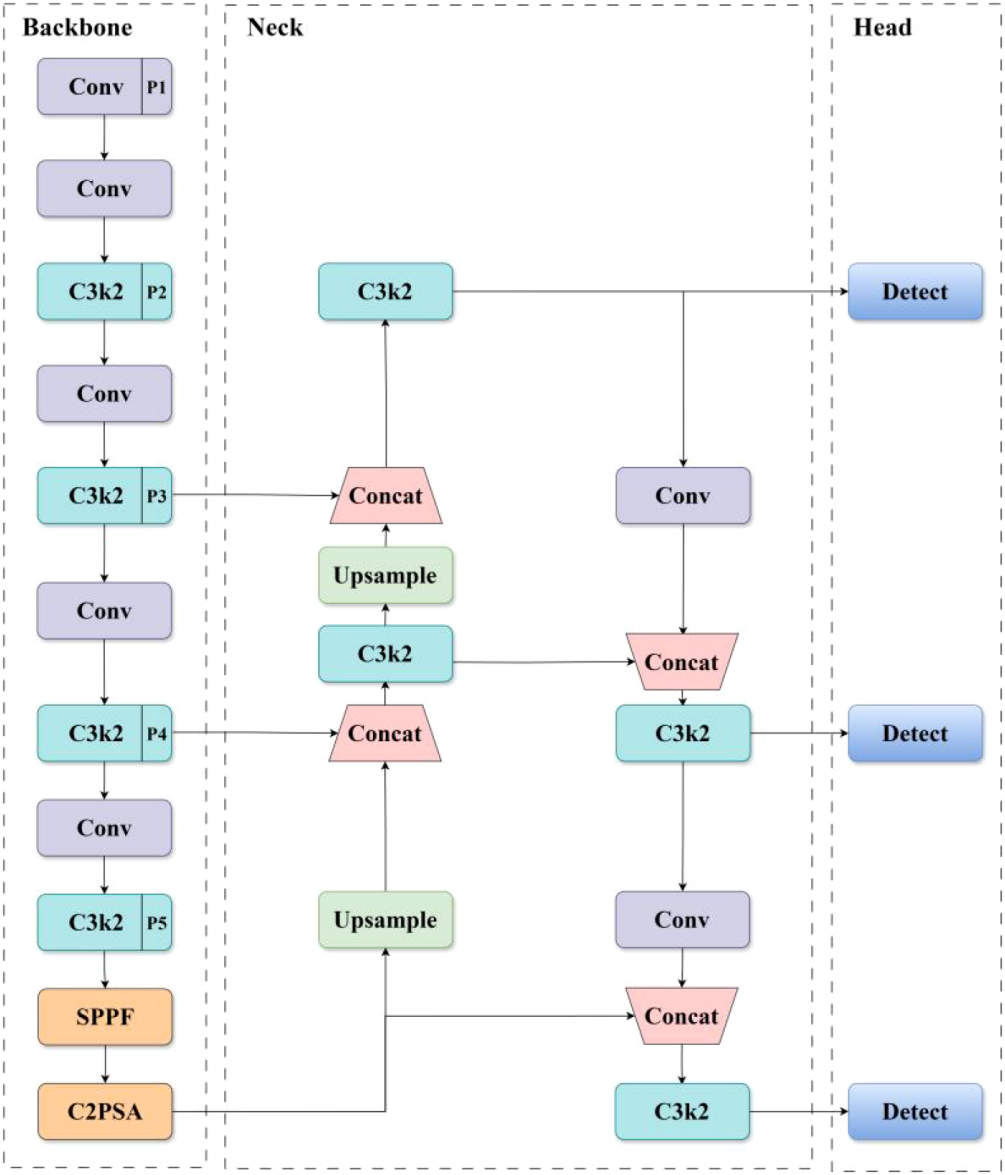
Network architecture of YOLOv11.

YOLOv11 (Khanam and Hussain, 2024) is the latest iteration of the YOLO (You Only Look Once) object detection model developed by Ultralytics. It offers five variants: Nano (n), Small (s), Medium (m), Large (l), and Extra-large (x), designed to meet different application requirements. Given the real-time performance and computational efficiency demands of inflorescence recognition tasks, YOLOv11s was adopted as the baseline model, with its network structure illustrated in **Figure 3**.

YOLOv11 consists of four main components: Input, Backbone, Neck and Head. The input module is responsible for image preprocessing, including data normalization and input size adjustment. The backbone network, which extracts image features, is primarily composed of Conv and C3k2 modules. The C3k2 module inherits the grouped convolution and cross-layer connection design from C2f (Varghese and Sambath, 2024), replacing the traditional 3×3 convolutional kernels with two 2×2 kernels, thereby significantly reducing computational complexity. The Conv module is composed of a convolutional layer, a batch normalization layer, and an activation function. However, due to its fixed receptive field size, it may overlook fine details when processing objects with significant scale variations.

The neck network employs a Path Aggregation Network (PAN) (Liu et al., 2018) structure, which improves multi-scale object detection accuracy by integrating low-level detail features with high-level semantic information through a top-down and bottom-up feature propagation mechanism. Finally, the detection head converts the feature maps from the neck network into final detection results using a decoupled head and an anchor-free design.

### 2.3 MDI-YOLOv11 model

**Figure 4:**
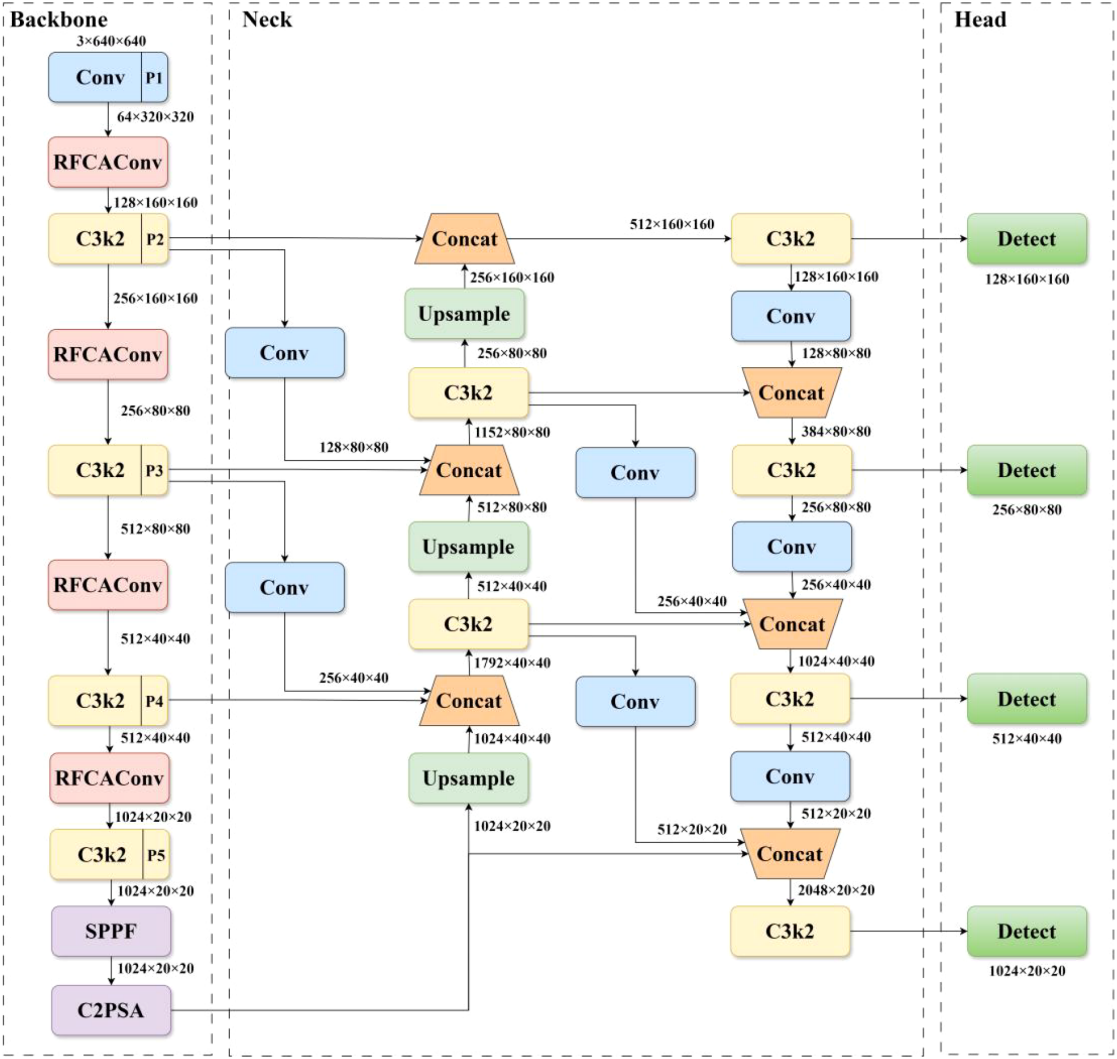
Network architecture of MDI-YOLOv11.

To achieve peach flower detection in natural scenes, an improved YOLOv11s model - MDI-YOLOv11 was proposed, which addressed the challenges posed by complex orchard environments, dense occlusion of peach flowers, and the high difficulty of bud recognition. The network architecture is illustrated in **Figure 4**.

In the backbone network, an RFCAConv module with attention mechanism (Zhang et al., 2023) is integrated to mitigate the insensitivity to spatial location information caused by parameter sharing in standard convolution, thus improving the model’s attention to critical regions while effectively addressing challenges posed by complex background interference and dense target occlusion. To address the issues of low accuracy in small object recognition and object information loss due to dense occlusion, the neck network utilizes an enhanced RepGFPN structure (Xu et al., 2022). By optimizing the feature pyramid construction process, deep semantic and shallow detail features are efficiently fused, the robustness of the model in multi-scale object detection is enhanced. Moreover, a P2 small object feature layer and corresponding small object detection head are incorporated to further improve the model’s capability to capture small object features.

#### 2.3.1 RFCAConv module

**Figure 5:**
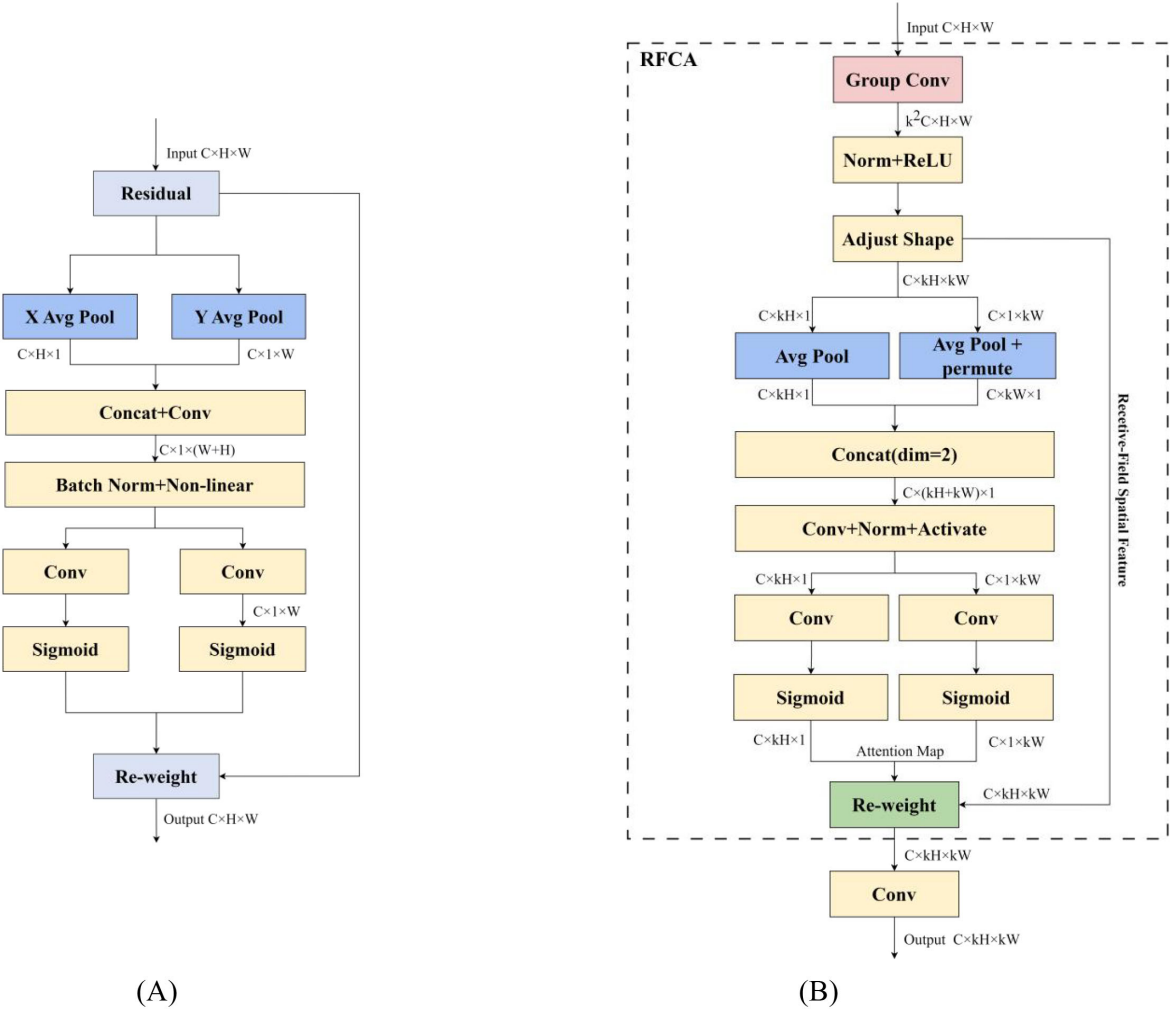
Structure of CA and RFCAConv modules: **(A)** CA module; **(B)** RFCAConv module.

To address the challenges of complex background interference and target occlusion in inflorescence detection, the model incorporated RFCAConv to replace standard convolution. RFCAConv extracts spatial receptive field features by grouped convolutions, and uses the coordinated attention (CA) (Hou et al., 2021) mechanism to dynamically reweight feature responses at different spatial locations according to their relative importance, thereby enhancing the model’s ability to emphasize inflorescence-related regions. The detailed structure is shown in **Figure 5B**.

**Figure 6:**
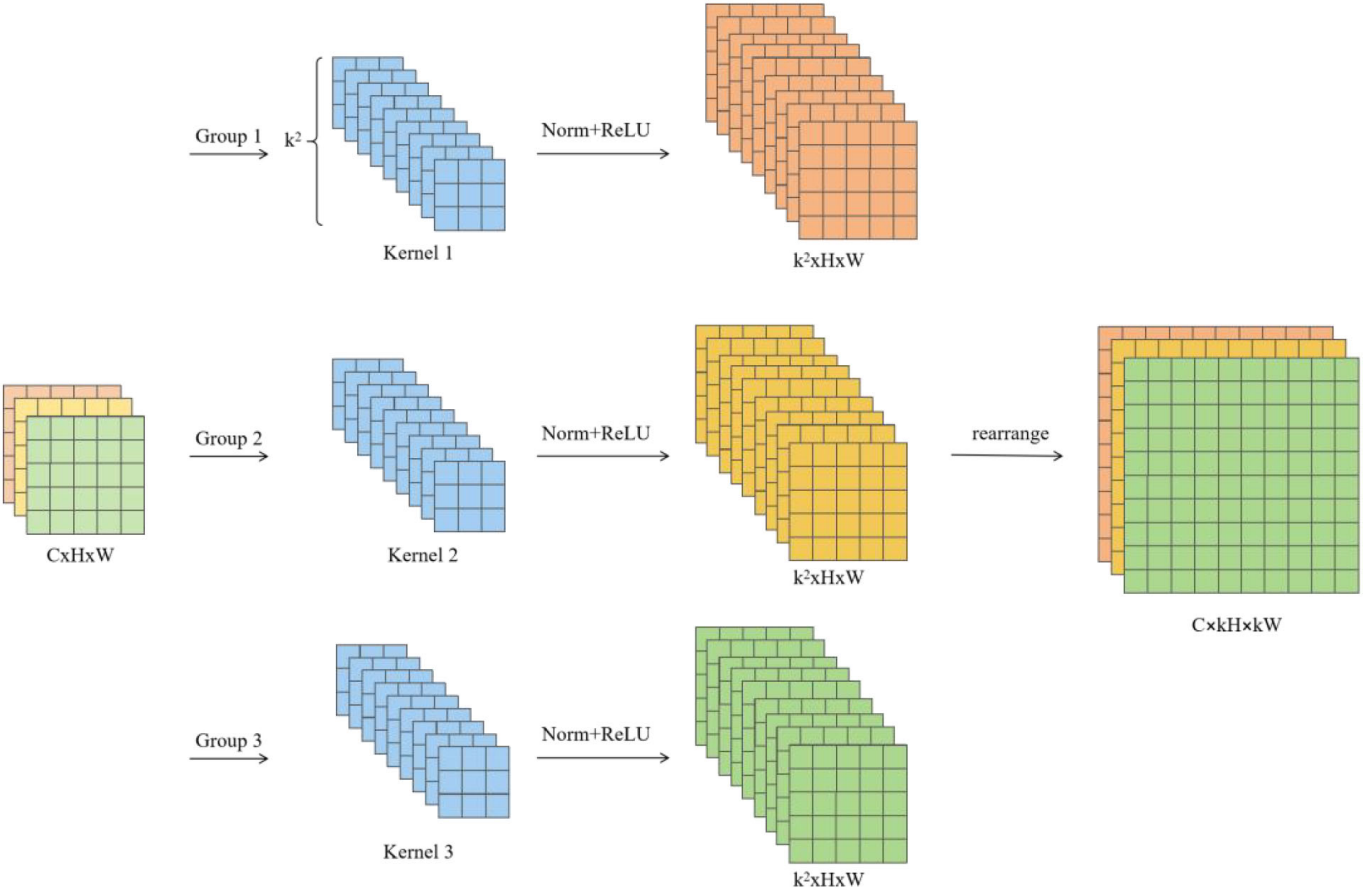
Process of receptive field feature map extraction.

In YOLOv11s, the standard convolution uses a shared convolution kernel to extract features, which means that the features at the corresponding positions in each sliding window share the same parameters, which makes it difficult for the model to capture the differences between different positions. The CA (**Figure 5A**) integrates spatial and channel attention to decouple the relationship between spatial information and channels. By encoding features separately along the horizontal and vertical directions, it generated direction-aware and position-sensitive attention weight maps. These maps were then applied to reweight the feature maps, assigning varying levels of importance to different regions, thereby mitigating the issue of parameter sharing within sliding windows. However, due to the overlap between sliding windows, the attention weight maps generated by the CA mechanism were still shared across these overlapping regions, failing to fully utilize the context information of the entire receptive field. The Receptive-field Attention Convolution (RFAConv) overcomes this limitation by employing grouped convolutions to project the original features into a wider receptive field, and then performing feature rearrangement to construct the receptive field representation, which facilitates the capture of relationships between local and global information (**Figure 6**). This receptive field feature map, composed of non-overlapping sliding windows, served as the input to the CA module to generate attention weight maps, effectively resolving the weight-sharing issue caused by overlapping sliding windows.

**Figure 7:**
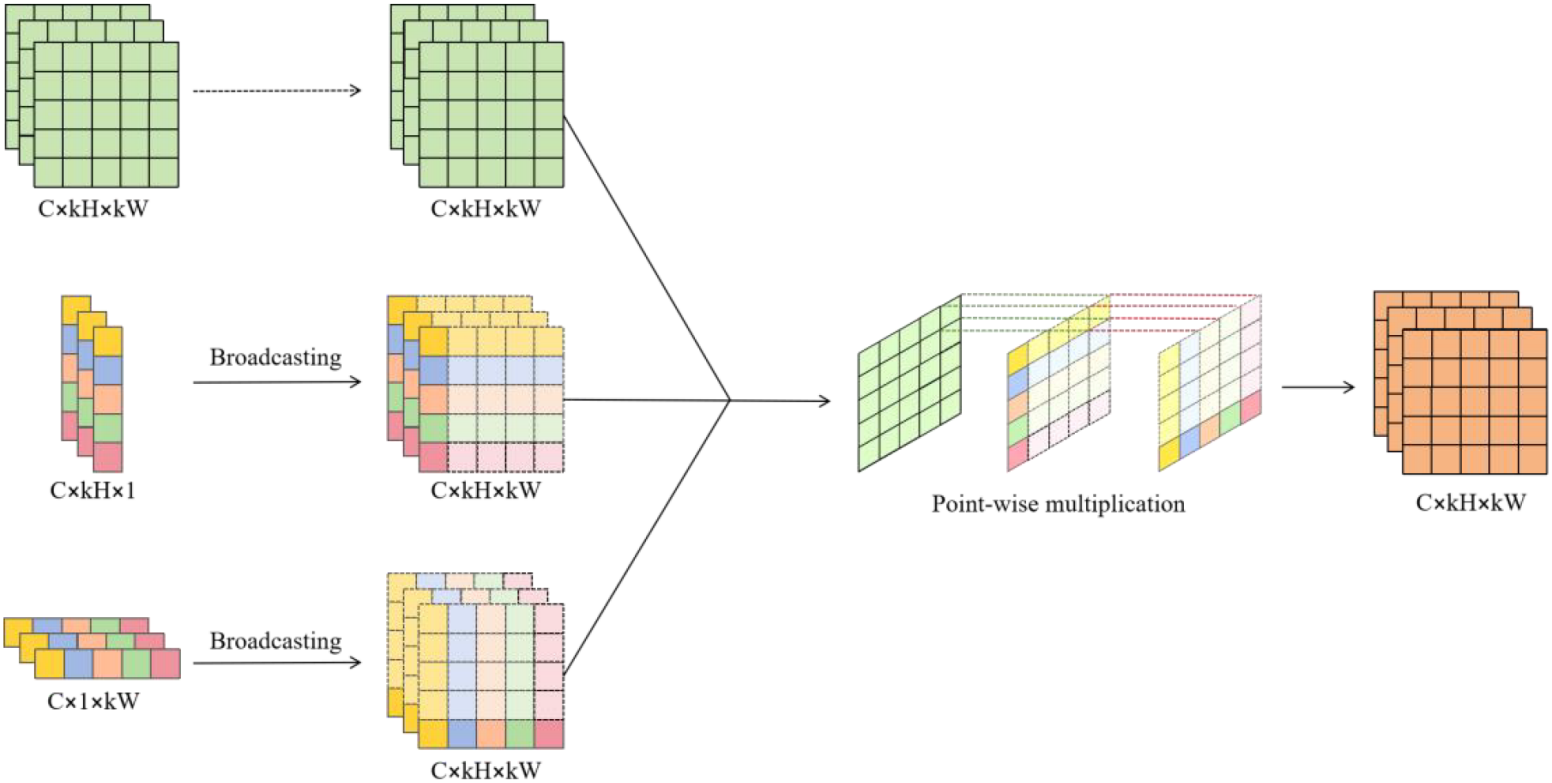
Re-weighting process.

**Figure 7** illustrates the detailed process of weighting the receptive field feature map, referred to as Re-weight. First, the attention weights extracted by the CA mechanism along both spatial and channel dimensions were broadcast to match the size of the receptive field feature map. Then, the broadcasted attention weight and the receptive field feature map are subjected to point-by-point product operation to obtain a weighted feature map. This weighting operation enhanced the model’s sensitivity to spatial position differences, enabling it to better focus on key areas of different spatial scales. Thus, the detection performance in scenarios with complex backgrounds, occlusions, and dense inflorescences is significantly improved.

Therefore, this study replaced the standard convolution in the backbone with the RFCAConv module. By thoroughly accounting for spatial variations across the entire feature map and assigning independent weights to each pixel, it effectively overcame the constraints imposed by parameter sharing in standard convolution.

#### 2.3.2 Efficient RepGFPN structure

**Figure 8:**
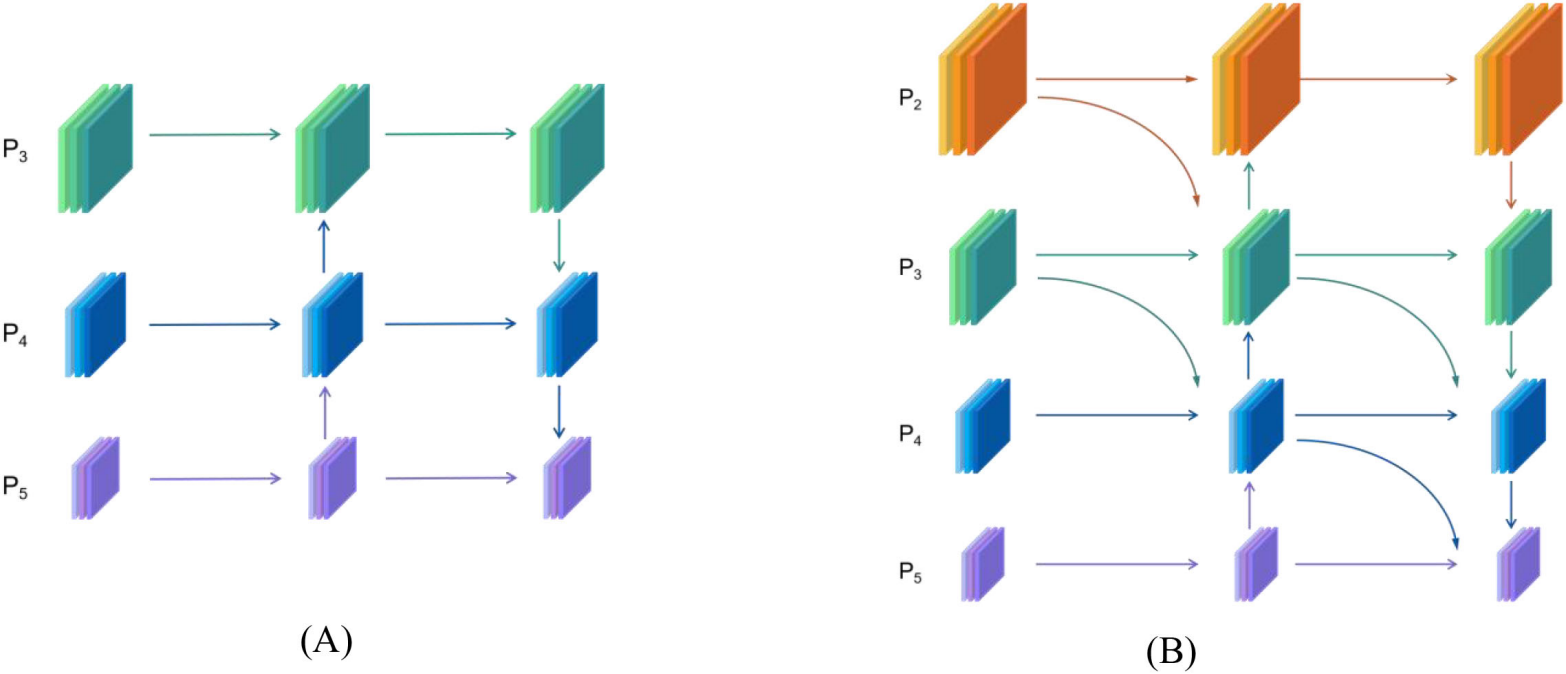
Design of the neck feature network: **(A)** PANet; **(B)** RepGFPN.

**Figure 9:**
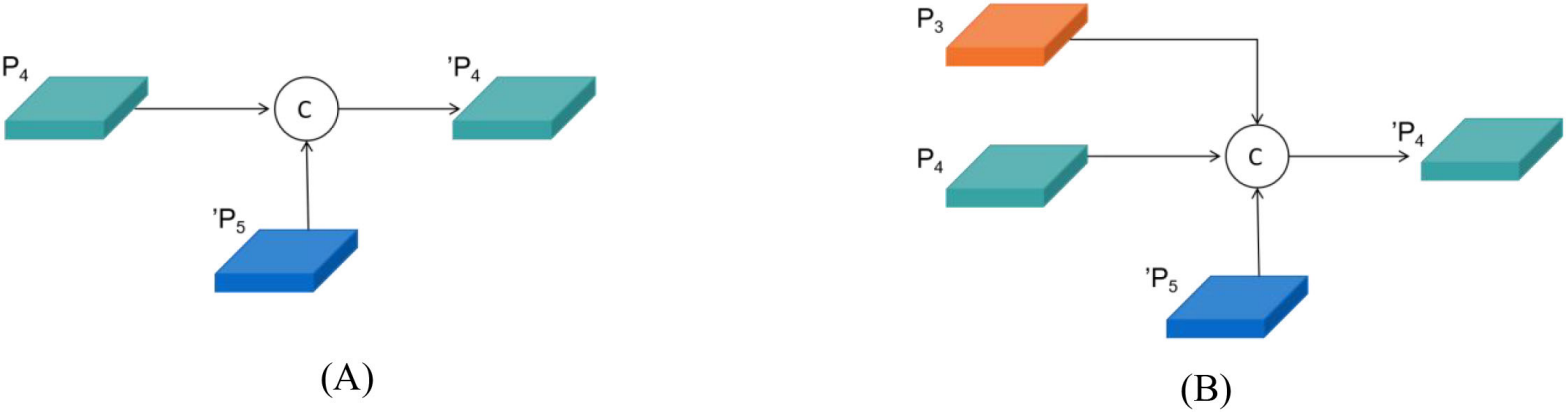
Comparison of cross-scale feature fusion between PANet and RepGFPN: **(A)** PANet; **(B)** RepGFPN.

In the Inflorescence recognition task, the targets in the image typically exhibit variations in size. Buds are usually small targets, while blossoms are large targets. However, the apparent size of these object is influenced by their relative distance: blossoms that are farther away may appear smaller in the image, whereas buds that are closer may appear relatively larger. This dynamic variation in size imposes higher demands on the model’s capacity for multi-scale feature extraction and fusion. YOLOv11 employed a PANet to fuse features at different scales, as illustrated in **Figure 8A**. It output three feature layers corresponding to feature maps of varying resolutions. Shallow, high-resolution feature maps preserved fine-grained edge and texture information, while deep, low-resolution feature maps retained abstract semantic and contextual information. By utilizing a bidirectional feature transfer mechanism that integrates top-down and bottom-up directions, PANet propagated deep semantic features to shallow layers and transmitted shallow detail features to deep layers, thereby enabling effective multi-scale feature aggregation. However, PANet only considered feature fusion within the same layer and from the preceding layer (as shown in **Figure 9A**), failing to fully retain fine-grained local information and neglecting the high-resolution features of the P2 layer.

To enhance the complementarity of multi-scale features and improve the model’s capability in detecting small objects, the RepGFPN structure was incorporated into the neck network of YOLOv11. Additionally, considering the detection requirements for small objects such as buds in inflorescence recognition, a P2 feature layer and a dedicated small-object detection head were introduced, as illustrated in **Figure 8B**. The RepGFPN adopted an improved Queen-Fusion structure (Jiang et al., 2022), which accounted for both intra-layer and inter-layer features during feature fusion. For example, within the P4 layer’s feature fusion process, the features from the preceding P3 layer were downsampled, and the features from the subsequent P5’ layer were upsampled before being concatenated with the current P4 layer (**Figure 9B**). This structure better balanced contextual and local information, enhancing both detection accuracy across multiple target scales and robustness in complex backgrounds. The newly added P2 feature layer represented higher resolution and finer image features, which helps to enhance the model’s ability to detect small targets and retain more detailed information. After incorporating the P2 layer into feature fusion, the improved Queen-Fusion structure effectively retained and enhanced these detailed features.

### 2.4 Model training and evaluation

#### 2.4.1 Experimental configuration

The model training environment was Ubuntu 22.04, with computational resources including an NVIDIA RTX 4090D GPU (24GB memory) and an 18-core AMD EPYC 9754 128-Core processor. The model was trained using Python 3.11 and the PyTorch 2.0.1 framework. Computational acceleration relies on CUDA 11.8 and cuDNN 8.5.0. Model training was conducted without pre-trained weights, using a single GPU. An early stopping strategy was employed to ensure effective convergence of each models. The specific training parameters are detailed in **Table 3**.

**Table 3:** Training parameters of the MDI-YOLOv11 model.

Training parameters	Values
Input Size	640×640
Initial learning rate	0.01
Batch size	8
Max epochs	500
Patience	20
Warm-up epochs	3
Weight decay	0.0005
Momentum	0.937

#### 2.4.2 Evaluation indicator

Six key metrics were employed to comprehensively evaluate the inflorescence detection model for peach trees, including average recall (AR_50_), average precision (AP_50_), detection speed, model size, total number of parameters, and computational complexity (GFLOPS). AP_50_ and AR_50_ have followed the MS COCO evaluation standards (Lin et al., 2014) to assess the model’s detection accuracy, representing the average precision and average recall at an Intersection over Union (IoU) threshold of 0.5, respectively. Detection speed, model size, parameter count, and computational complexity are employed to evaluate the efficiency and computational requirements of the model. Detection speed indicates the time required to process each image, reflecting the model’s inference efficiency. The total parameter count represents the trainable parameters in the model, GFLOPS represents the number of floating-point operations per second, which is used to measure the computational complexity of the model, and the model size indicates the storage requirement for the model’s weight file.

Precision and recall (Everingham et al., 2010) are calculated using the following formulas:


$$Precision=\frac{TP}{TP+FP}$$

$$Recall=\frac{TP}{TP+FN}$$

Where, True Positive (*TP*) refers to cases where the IoU between the predicted box and the ground-truth box is ≥0.5. False Positive (*FP*) refers to cases where the IoU between the predicted box and the ground-truth box is <0.5. False Negative (*FN*) refers to cases where the model fails to predict the corresponding target box.

## 3 Results

### 3.1 Ablation experiment

To thoroughly analyze the functional contributions of each module in the MDI-YOLOv11 model, YOLOv11s was selected as the baseline model, and ablation experiments were conducted on the peach tree inflorescence dataset using the same data splits. The results are shown in **Table 4**.

**Table 4:** Evaluation result of the ablation experiment.

Basic network	P2GFPN	RFCAConv	Cross validation	All		Blosssom		Bud	
				AP_50_	AR_50_	AP_50_	AR_50_	AP_50_	AR_50_
YOLOv11s			Fold1	0.902	0.941	0.916	0.966	0.888	0.916
			Fold2	0.885	0.933	0.891	0.955	0.880	0.911
			Fold3	0.874	0.919	0.898	0.957	0.849	0.880
			Fold4	0.890	0.935	0.913	0.966	0.868	0.904
			Fold5	0.881	0.925	0.909	0.961	0.853	0.888
			**Average**	**0.886**	**0.931**	**0.905**	**0.961**	**0.868**	**0.900**
	✓		Fold1	0.919	0.961	0.926	0.980	0.913	0.943
			Fold2	0.909	0.960	0.903	0.969	0.915	0.952
			Fold3	0.899	0.954	0.910	0.981	0.888	0.928
			Fold4	0.916	0.965	0.921	0.979	0.911	0.951
			Fold5	0.912	0.958	0.922	0.979	0.901	0.938
			**Average**	**0.911**	**0.960**	**0.916**	**0.978**	**0.906**	**0.942**
		✓	Fold1	0.905	0.939	0.919	0.963	0.892	0.914
			Fold2	0.891	0.932	0.898	0.954	0.884	0.910
			Fold3	0.883	0.925	0.907	0.964	0.859	0.887
			Fold4	0.891	0.932	0.916	0.964	0.867	0.900
			Fold5	0.893	0.930	0.919	0.965	0.866	0.895
			**Average**	**0.893**	**0.932**	**0.912**	**0.962**	**0.874**	**0.901**
	✓	✓	Fold1	0.928	0.967	0.933	0.981	0.924	0.953
			Fold2	0.914	0.963	0.909	0.972	0.920	0.955
			Fold3	0.912	0.958	0.921	0.982	0.902	0.934
			Fold4	0.925	0.970	0.933	0.984	0.917	0.956
			Fold5	0.916	0.962	0.924	0.980	0.908	0.944
			**Average**	**0.919**	**0.964**	**0.924**	**0.980**	**0.914**	**0.948**

Bolded values indicate the average performance obtained from five-fold cross-validation.

According to **Table 4**, the baseline model YOLOv11s attains AP_50_ values of 0.905 and 0.868 for the blossom and bud categories, respectively, and AR_50_ values of 0.961 and 0.9, respectively. The average AP_50_ is 0.886, and the average AR_50_ is 0.931. The results indicate that the overall detection accuracy is relatively low, especially for the bud category. By incorporating the RFCAConv module and the P2GFPN structure to optimize the model, the improved MDI-YOLOv11 model increased its AP_50_ and AR_50_ by 0.033 compared to the baseline model, reaching 0.919 and 0.964, respectively. Specifically, for the flower category, both AP_50_ and AR_50_ improved by 0.019, while for the bud category, AP_50_ and AR_50_ increased by 0.046 and 0.048.

**Figure 10:**
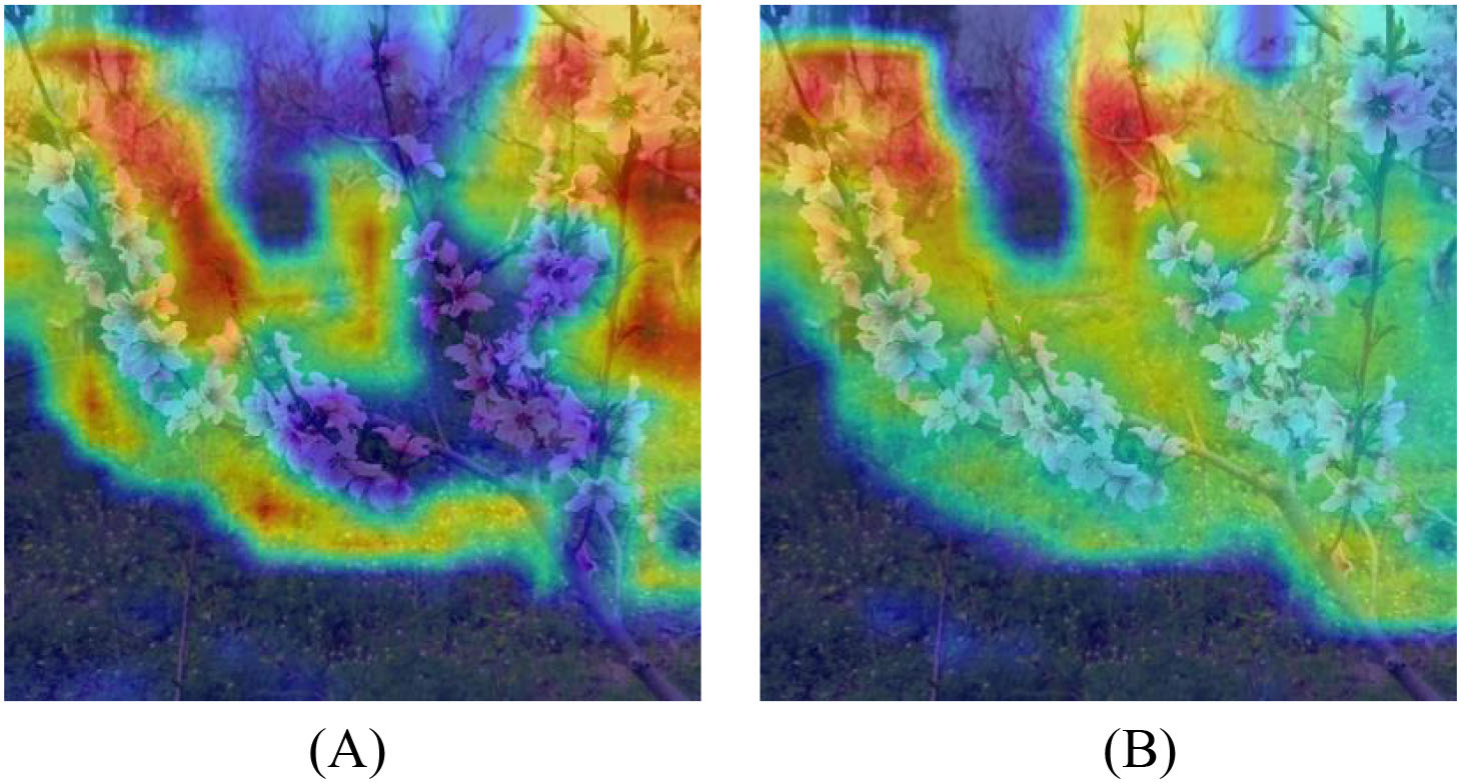
Visual comparison results of the RFCAConv module: **(A)** Without RFCAConv; **(B)** With RFCAConv.

When the RFCAConv module was incorporated alone, AP_50_ increased by 0.007, and AR_50_ increased by 0.001. Although the improvement in model performance was modest, the RFCAConv module effectively directed the model’s attention toward the inflorescence regions, enhancing its robustness against interference from complex backgrounds. **Figure 10** presents the heatmap comparison of the backbone output layer before and after the integration of the RFCAConv module. In **Figure 10A**, the heatmap of the baseline model shows weak focus on the target regions and excessive attention to background areas. After the integration of the RFCAConv module, the heatmap illustrates a more concentrated focus on the inflorescence regions (**Figure 10B**), indicating an enhanced capability of the network to recognize targets under complex background conditions.

**Figure 11:**
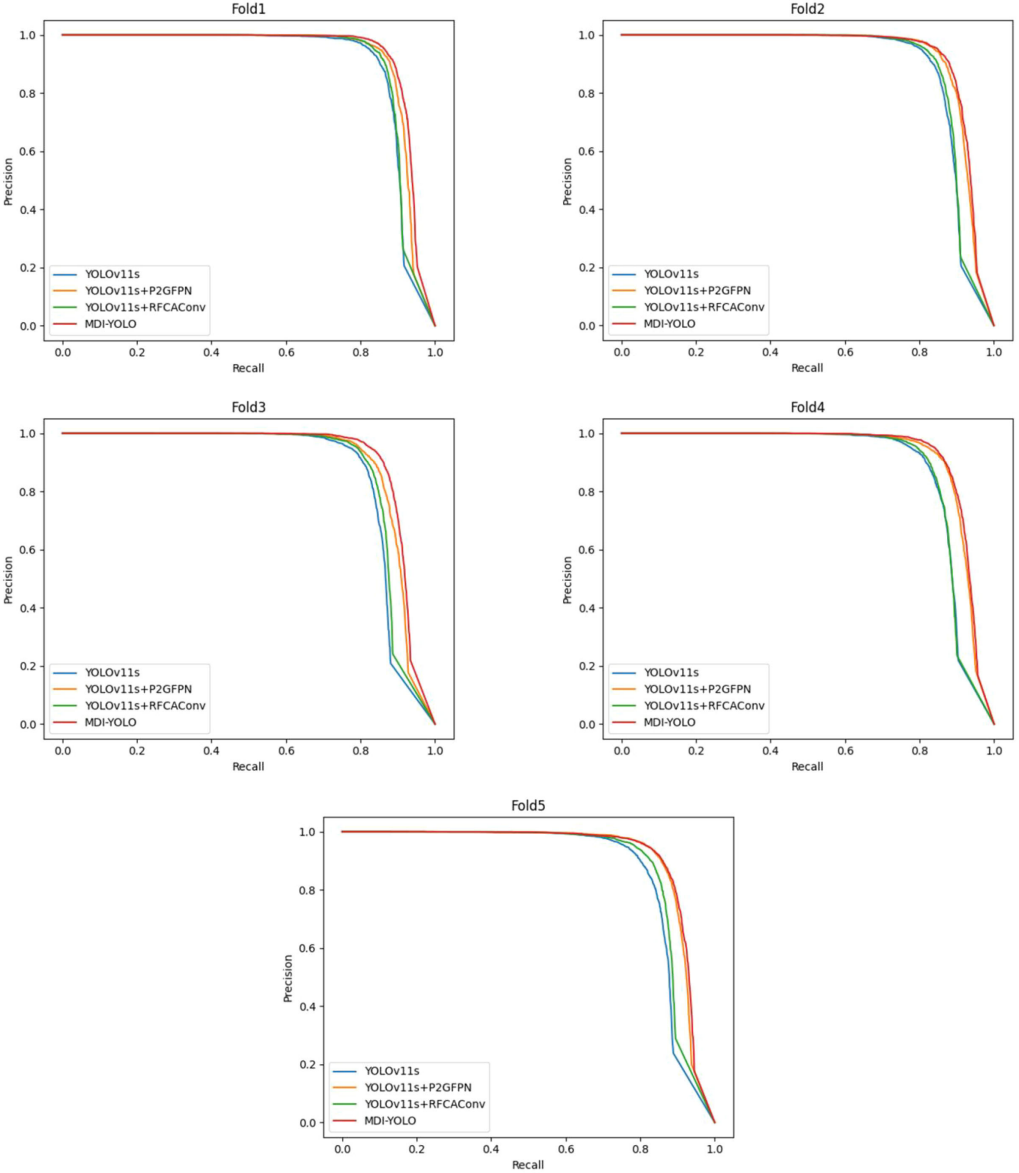
The P-R curves for the bud category in ablation experiments.

Compared to merely adding the RFCAConv module, the P2GFPN structure achieves a more pronounced improvement in YOLOv11s, particularly in the detection performance for the flower bud category. **Figure 11** presents the comparison of P-R curves for the flower bud category in the ablation experiment, which intuitively illustrates the detection performance of different models on flower bud targets. As depicted in **Figure 11**, the first half of the four curves has an accuracy close to 1, indicating that the models are highly accurate in detecting flower buds at low recall rates, with minimal false detections. As recall increases, the accuracy of the YOLOv11s curve drops first, suggesting that this model struggled to effectively control false positives when recall was increased, leading to a higher number of false detections. The YOLOv11s + RFCAConv curve shows some improvement over the YOLOv11s curve when recall increases, but the two curves almost overlap at high recall rates, indicating that the RFCAConv module had limited optimization effect on false positives. In contrast, the YOLOv11s + P2GFPN curve is significantly higher and more to the right, and is very close to the MDI-YOLOv11 curve, indicating that the P2GFPN structure was instrumental in improving the detection of flower bud targets. In terms of performance metrics, the overall AP_50_ and AR_50_ increase by 0.025 and 0.029, respectively. For the blossom category, AP_50_ and AR_50_ increase by 0.011 and 0.017, respectively, while for the flower bud category, AP_50_ and AR_50_ increase by 0.038 and 0.042, respectively. These results indicate that the P2GFPN structure not only enhances the detection performance of small targets but also boosts the model’s overall performance in multi scale inflorescence detection.

**Figure 12:**
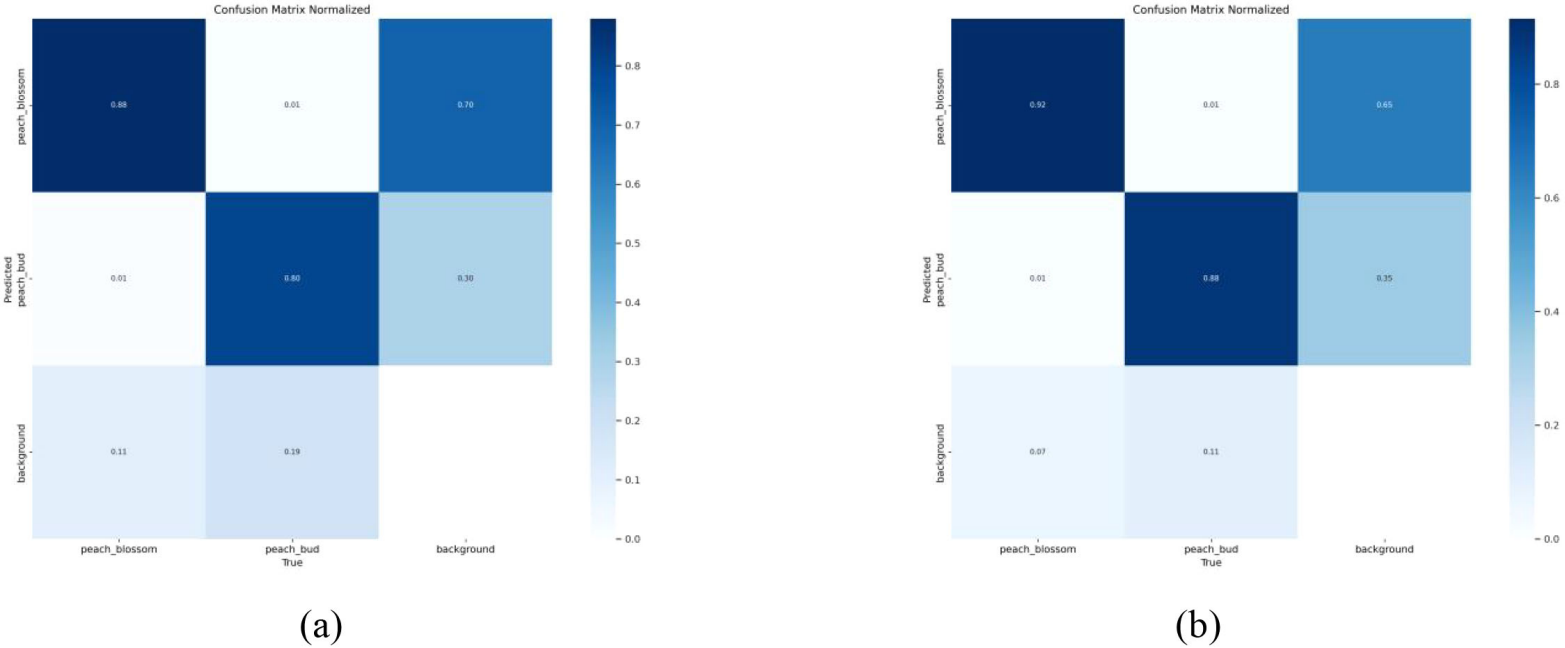
Confusion matrix comparison between YOLOv11s and MDI-YOLOv11: **(a)** Confusion matrix of YOLOv11s; **(b)** Confusion matrix of MDI-YOLOv11.

To quantitatively evaluate the effectiveness of the model optimization from the perspective of classification performance, **Figure 12** further presents a comparison of the normalized confusion matrices before and after model improvement. In the confusion matrices, the horizontal axis represents the ground truth labels, while the vertical axis denotes the predicted labels. The values along the main diagonal correspond to the classification accuracy of each category, while the elements outside the diagonal reflect misclassification and missed detection between classes. As shown in **Figure 12**, the baseline YOLOv11s model achieves classification accuracies of 88% and 80% for flowers and buds, respectively, with corresponding missed detection rates of 11% and 19%. In contrast, the improved MDI-YOLOv11 model exhibits substantial improvements in both metrics, with classification accuracies increased to 92% and 88%, and missed detection rates reduced to 7% and 11%, respectively. These results indicate that the proposed optimization mechanisms not only improve the model’s discriminative capability between visually similar classes, but also effectively improve the recall performance for foreground targets, further validating the effectiveness of the proposed model improvements.

### 3.2 Performance comparison of different models

**Figure 13:**
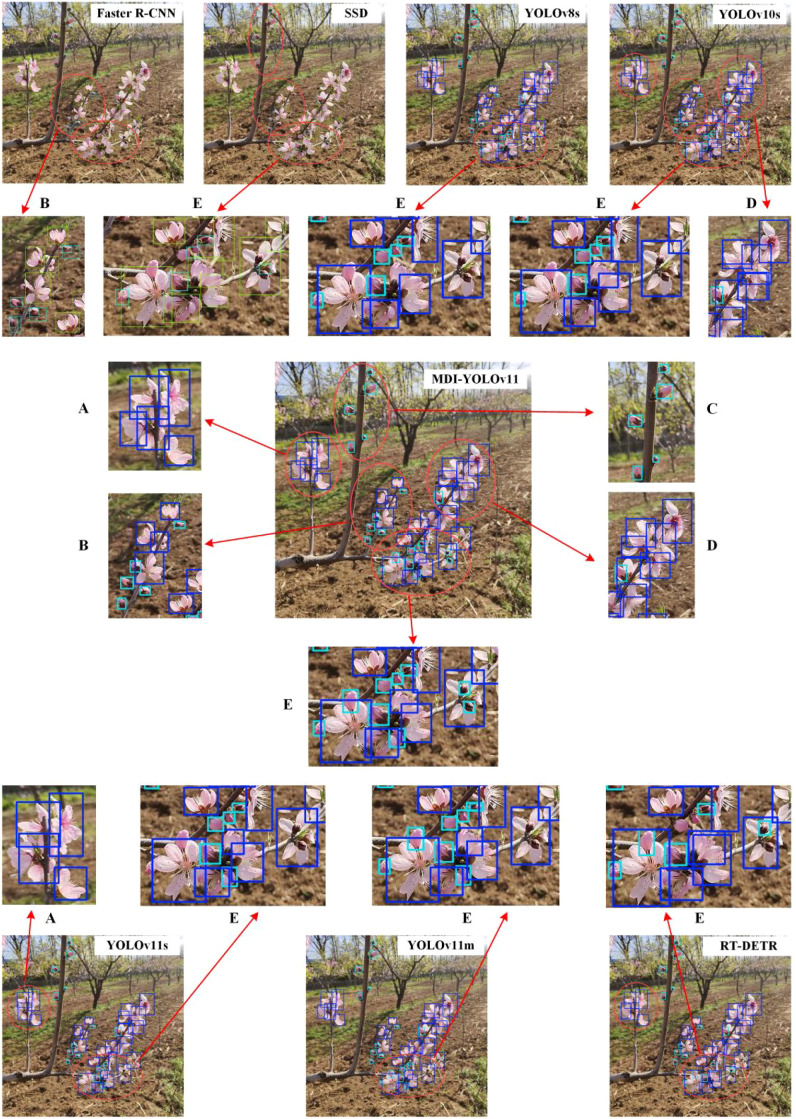
Visual detection results of comparative models: Regions **A–E** indicate different areas within the detection image.

To validate the performance of the proposed MDI-YOLOv11 model, a comparison was conducted with seven other object detection models: Faster R-CNN (Ren et al., 2016), SSD (Liu et al., 2016), RT-DETR (Zhao et al., 2024), YOLOv8s (Varghese and Sambath, 2024), YOLOv10s (Wang et al., 2024), YOLOv11m, and the baseline model YOLOv11s. The models encompassed classical object detection approaches (such as Faster R-CNN and SSD), the recently popular RT-DETR model, and the v8 and v10 versions of the YOLO series. Additionally, YOLOv11m is included to verify that the improved model outperforms YOLOv11m in accuracy while maintaining a more favorable balance between parameter size and computational complexity. All comparison models were trained using the same data partitioning. The detection results are shown in **Tables 5**, **6** and **Figure 13**.

**Table 5:** Results of detection performance with different models.

Models	Cross validation	AP_50_	AR_50_	Models	Cross validation	AP_50_	AR_50_
Faster R-CNN	Fold1	0.672	0.704	YOLOv10s	Fold1	0.893	0.936
	Fold2	0.663	0.699		Fold2	0.874	0.930
	Fold3	0.635	0.665		Fold3	0.871	0.921
	Fold4	0.659	0.697		Fold4	0.891	0.939
	Fold5	0.647	0.680		Fold5	0.876	0.927
	**Average**	**0.655**	**0.689**		**Average**	**0.881**	**0.931**
SSD	Fold1	0.615	0.683	YOLOv11s	Fold1	0.902	0.941
	Fold2	0.606	0.684		Fold2	0.885	0.933
	Fold3	0.589	0.658		Fold3	0.874	0.919
	Fold4	0.602	0.672		Fold4	0.890	0.935
	Fold5	0.598	0.665		Fold5	0.881	0.925
	**Average**	**0.602**	**0.672**		**Average**	**0.886**	**0.931**
RT-DETR	Fold1	0.882	0.922	YOLOv11m	Fold1	0.916	0.945
	Fold2	0.908	0.943		Fold2	0.902	0.938
	Fold3	0.888	0.926		Fold3	0.896	0.930
	Fold4	0.904	0.939		Fold4	0.911	0.943
	Fold5	0.881	0.923		Fold5	0.907	0.940
	**Average**	**0.893**	**0.931**		**Average**	**0.906**	**0.939**
YOLOv8s	Fold1	0.898	0.936	MDI-YOLOv11	Fold1	0.928	0.967
	Fold2	0.881	0.929		Fold2	0.914	0.963
	Fold3	0.866	0.916		Fold3	0.912	0.958
	Fold4	0.892	0.935		Fold4	0.925	0.970
	Fold5	0.881	0.922		Fold5	0.916	0.962
	**Average**	**0.884**	**0.928**		**Average**	**0.919**	**0.964**

Bolded values indicate the average performance obtained from five-fold cross-validation.

**Table 6:** Detection efficiency indicators with different models.

Method	Total params (M)	Model size (MB)	Speed (ms/image)
Faster R-CNN	82.36	628.56	9.4
SSD	13.19	101.19	4.7
RT-DETR	22.20	45.85	26.6
YOLOv8s	11.14	21.51	6.14
YOLOv10s	8.07	15.82	9.38
YOLOv11s	9.43	18.32	8.05
YOLOv11m	20.06	38.67	9.92
MDI-YOLOv11	10.97	21.51	13.46

**Tables 5**, **6** demonstrate that the MDI-YOLOv11 model achieves the highest AP_50_ and AR_50_. In comparison to Faster R-CNN, YOLOv8s, YOLOv10s, YOLOv11s, SSD, RT-DETR, and YOLOv11m, the AP_50_ of MDI-YOLOv11 is higher by 0.264, 0.317, 0.026, 0.035, 0.038, 0.033, and 0.013, respectively, while the AR_50_ shows improvements of 0.275, 0.292, 0.033, 0.036, 0.033, 0.033, and 0.025, respectively. These enhancements significantly outperform the other models. The MDI-YOLOv11 model has 10.97M parameters and a model size of 21.51MB, both smaller than those of SSD, RT-DETR, Faster R-CNN, YOLOv8s, and YOLOv11m, but slightly larger than those of YOLOv10s and YOLOv11s. Although MDI-YOLOv11 sacrifices some detection speed, it still maintains a processing speed of 13.46 milliseconds per image, which remains sufficient for real-time detection. Moreover, the higher accuracy is more beneficial to the inflorescence detection tasks. Compared with YOLOv11m from the same series, MDI-YOLOv11 is approximately half the size and complexity, yet its AP_50_ improves by 0.013 and AR_50_ by 0.025, achieving a better balance between accuracy and efficiency.

**Figure 13** presents the visual detection results of MDI-YOLOv11 and the comparison models. In Region E, where target overlap is high, all comparison models demonstrate missed detections of flower buds to varying extents. In particular, the two flower buds on the far right of Region E that are superimposed above a flower are not accurately detected by any of the comparision models. In contrast, MDI-YOLOv11 successfully and precisely locates all objects. Faster R-CNN performes poorly, with significant missed detections and inaccurate bounding box localization. For instance, in the lower and upper right parts of Region B, Faster R-CNN generates two bounding boxes for a single flower bud, a phenomenon confirmed to be independent of the threshold setting. YOLOv10s and YOLOv11s exhibited suboptimal performance in handling densely arranged adjacent flowers. YOLOv10s incorrectly identifies the same flower multiple times in the middle of Region D, while YOLOv11s fail to detect an unobstructed flower located in the left-central part of Region A. Although YOLOv8s and YOLOv11m demonstrate relatively better localization accuracy, they show insufficient sensitivity to flower buds and, compare to the MDI-YOLOv11 model, still exhibit certain limitations in handling overlapping, occluded, and densely arranged targets.

In summary, the MDI-YOLOv11 model performs exceptionally well in the peach tree inflorescence detection task, achieving AP_50_ and AR_50_ of 0.919 and 0.964, respectively. With only 10.97M parameters, the model maintains a good balance between detection performance and model complexity, and can effectively deal with the real-time detection task of inflorescence in complex scenes. The algorithm not only surpasses other object detection models in terms of accuracy but also demonstrates superior adaptability in handling densely occluded targets and higher precision in detecting small targets. It meets the dual requirements of real-time performance and high accuracy for orchard inflorescence detection.

### 3.3 Inflorescence density mapping

**Figure 14:**
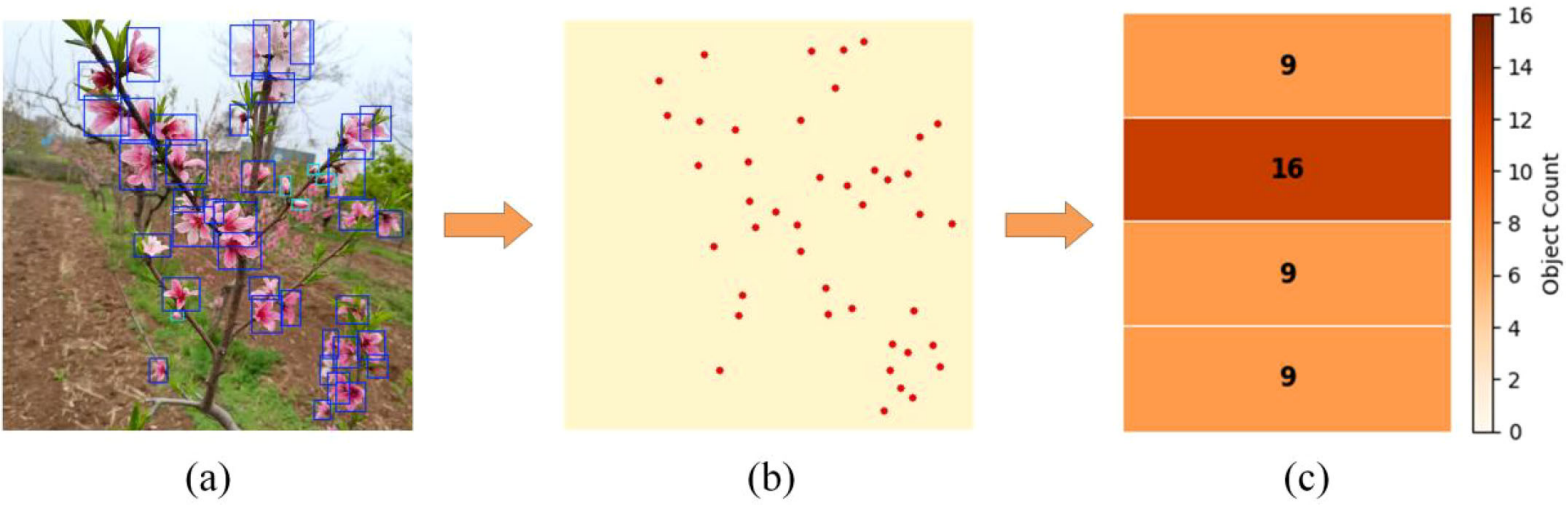
Inflorescence density mapping: **(a)** Inflorescence detection results; **(b)** Distribution map of target center points; **(c)** Inflorescence density distribution map. The numbers in each row of (c) indicate the count of floral targets within that row region.

To more clearly illustrate the density and spatial distribution of peach tree inflorescences, a row-based inflorescence density distribution heatmap was developed based on the detection results, as illustrated in **Figure 14**.

**Figure 14a** presents the inflorescence detection results obtained using the MDI-YOLOv11 model. **Figure 14b** displays the distribution map of bounding box centers derived from the detection results. **Figure 14c** presents a row-wise density distribution map generated by combining the detection results in **Figure 14a** and the center-point distribution in **Figure 14b**. Specifically, the image is divided into four horizontal regions, and the number of targets within each region is calculated based on the spatial distribution of the bounding box centroids. Then, the resulting regional inflorescence density distribution is visualized in the form of a heat map, enabling an intuitive representation of spatial variations in inflorescence distribution.

As shown in **Figure 14c**, the distribution of flowers within different regions of the same peach tree exhibits pronounced spatial heterogeneity, reflecting the inherent variability of peach flower distribution under natural growth conditions. In practical orchard management, this sub-region inflorescence density map can provide direct guidance for differentiated flower-thinning strategies. For instance, higher thinning intensity may be applied in regions with high inflorescence density, whereas thinning intensity or operation duration can be reduced in low-density regions to avoid over-thinning. This approach can provide data support for the formulation of flower thinning strategies, and help to effectively connect the inflorescence recognition results with the subsequent intelligent flower thinning operations. Furthermore, the number of density map partitions can be flexibly adjusted according to different tree architectures and specific operational requirements, allowing the generation of inflorescence density distributions that better reflect real orchard conditions. Without increasing the computational complexity of the model, this spatial distribution visualization based on detection results provides an intuitive and reliable decision-making reference for intelligent flower thinning and automated orchard management.

## 4 Discussion

### 4.1 Analysis of inflorescence recognition results by MDI-YOLOv11

Peach inflorescences exhibit pronounced morphological variations across different flowering stages and are typically densely distributed with frequent mutual occlusion. In addition, flower buds are relatively small and may be similar to petals under side view conditions. These characteristics impose stringent requirements on peach flower detection in natural orchard environments. In practical orchard operation scenarios, complex and dynamic lighting conditions can lead to uneven brightness distribution and shifts in color features, thereby significantly affecting the stability of feature extraction and classification in visual perception models. Such illumination variability constitutes one of the key external factors influencing model performance (Yunchao et al., 2023).

**Figure 15:**
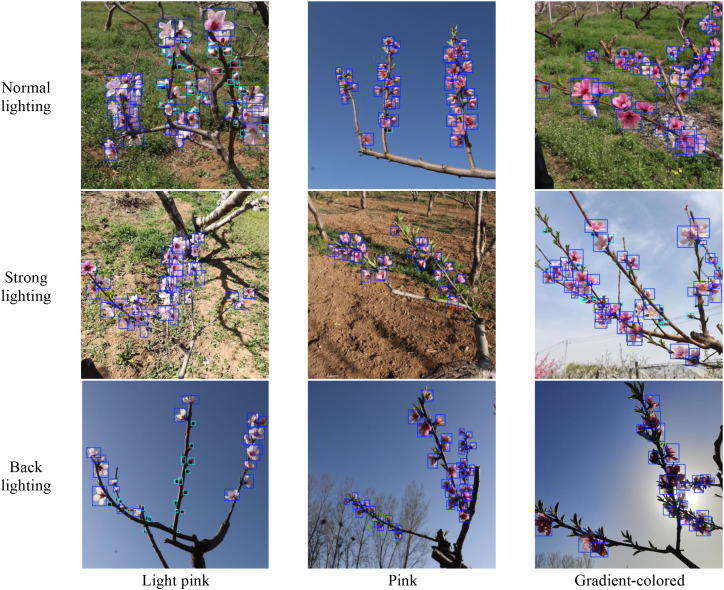
Example detection results of flowers in different colors under different lighting conditions.

For the evaluation of the MDI-YOLOv11 model’s detection capability in complex conditions, the dataset used in this study covers three typical illumination scenarios(normal lighting, backlighting, strong lighting) and three flower color types(light pink, pink, gradient-colored). During model training, samples from different illumination and color conditions were jointly modeled to improve the model’s robustness under complex orchard conditions. The detection results under different lighting and color conditions are shown in **Figure 15**.

The MDI-YOLOv11 model is able to stably identify the categories and spatial locations of peach flowers with different colors even under challenging lighting conditions such as strong lighting and backlighting. This demonstrates that the model has certain robustness to illumination variations. This superior performance was primarily attributed to improvements in the backbone and neck networks. The introduction of the RFCAConv module in the backbone network enhanced the model’s ability to focus on key contextual information of peach tree inflorescences, improving feature extraction and robustness against interference while maintaining low computational overhead. In the neck network, the P2 small-object feature layer effectively preserved fine details from shallow feature maps, while the improved Queen-Fusion structure facilitated the integration of cross-layer semantic and detailed features, further enhancing detection performance.

### 4.2 Comparative analysis of model recognition performance

This study constructed a peach tree inflorescence detection model, MDI-YOLOv11, using a self-constructed peach tree inflorescence dataset, achieving an AP_50_ of 0.919 and an AR_50_ of 0.964. In contrast, Shang et al. (Yuying et al., 2023) collected 3005 images of apple inflorescences and constructed a lightweight real-time detection model with a size of only 0.61 MB, facilitating its deployment on edge devices. Their detection accuracy is merely 0.001 lower than ours, mainly because their dataset consisted of close-up images of inflorescences, which provides clearer target features, and because the dataset size is larger. Compared to MTYOLOX (Xue et al., 2023), the proposed approach achieved improvements of 0.085 and 0.031 in AP_50_ and AR_50_, respectively. This can be attributed to the fact that our dataset consists of localized large-branch peach inflorescences, whereas MTYOLOX is trained on whole-tree inflorescences, which pose greater detection challenges. Future research will incorporate whole-tree inflorescence data to expand the dataset and explore model lightweighting to enhance adaptability across different application scenarios.

In addition, the dataset constructed in this study covers diverse illumination conditions, different flower color varieties, and a wide range of orchard backgrounds, which enhances the model’s adaptability to variations in real orchard environments to a certain extent. From the perspectives of model architecture design and data diversity, the proposed MDI-YOLOv11 model does not rely on a specific peach variety and therefore exhibits potential applicability to other peach varieties as well as to inflorescence recognition tasks of similar stone fruit tree species. In future work, public fruit tree inflorescence datasets and data collected from different regions and varieties will be further incorporated to conduct a more comprehensive evaluation of the model’s cross-dataset and cross-variety generalization performance, thereby further improving its practical value in orchard management.

### 4.3 Analysis of peach flower missed detection

**Figure 16:**
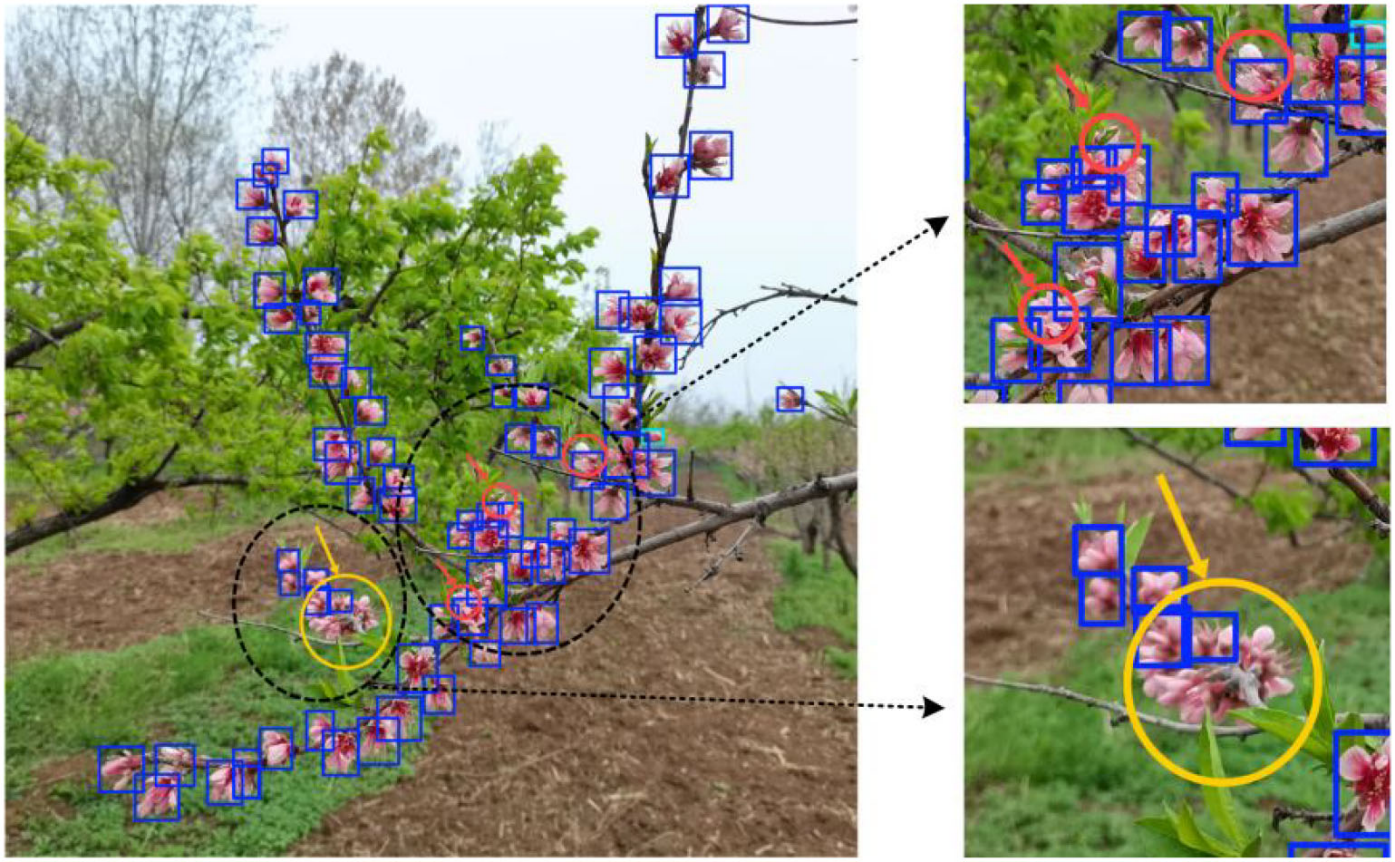
Example of missed detection in peach flowers.

Although the proposed method improves detection performance under conditions of dense occlusion, intelligent flower-thinning machines still face the following challenges during operation in real orchard environments: (1) heavily occluded targets are difficult to accurately identify; (2) distant targets may be too small or blurred, making detection unreliable. As shown in **Figure 16**, the flowers indicated by red arrows are largely obscured by other flowers and remain undetected, while those marked by yellow arrows are densely arranged and blurred due to motion, resulting in partial detection failure.

To address these issues, integrating depth information will be an effective solution. By using depth information (Tan et al., 2024) to remove the background and focused on target detection in a specific depth plane, it will help to solve the problem that distant small targets cannot be recognized. For heavily occluded targets, detection and thinning will be performed again in the next depth plane.

## 5 Conclusion

In order to realize the accurate and real-time detection of peach inflorescences in the actual orchard scene, this study introduces an improved YOLOv11s algorithm, termed MDI-YOLOv11, to address detection challenges arising from a high proportion of small targets, severe occlusion, and scale variation. Experimental results demonstrate that MDI-YOLOv11 exhibits strong robustness under complex illumination conditions and dense inflorescence scenarios. Compared with SSD, RT-DETR, Faster R-CNN, YOLOv8s, YOLOv10s, and YOLOv11m, MDI-YOLOv11 achieves a better balance between detection accuracy and model complexity, with an AP_50_ of 0.919 and an AR_50_ of 0.964, a single image detection time of 13.46ms, and a model size of 21.51MB. Compared to the baseline model YOLOv11s, AP_50_ and AR_50_ are improved by 0.033. Based on the detection results of MDI-YOLOv11, the heatmap of inflorescence density distribution are further generated to visually present spatial variations in inflorescence distribution, providing supportive spatial information for precision flower thinning in orchards. Future work will focus on exploring the model’s adaptability across different varieties, three-dimensional spatial positioning, and real-time integration with orchard management systems, thereby enhancing its practical value in large scale orchards.

## Data Availability

The raw data supporting the conclusions of this article will be made available by the authors, without undue reservation.
